# Building resource-efficient community databases using open-source software

**DOI:** 10.1093/database/baaf005

**Published:** 2025-02-12

**Authors:** Sook Jung, Chun-Huai Cheng, Taein Lee, Katheryn Buble, Jodi Humann, Ping Zheng, Jing Yu, Dorrie Main

**Affiliations:** Department of Horticulture, Washington State University, 303c Plant Sciences Building, Pullman, WA 99164-6414, USA; Department of Horticulture, Washington State University, 303c Plant Sciences Building, Pullman, WA 99164-6414, USA; Department of Horticulture, Washington State University, 303c Plant Sciences Building, Pullman, WA 99164-6414, USA; Department of Horticulture, Washington State University, 303c Plant Sciences Building, Pullman, WA 99164-6414, USA; Department of Horticulture, Washington State University, 303c Plant Sciences Building, Pullman, WA 99164-6414, USA; Department of Horticulture, Washington State University, 303c Plant Sciences Building, Pullman, WA 99164-6414, USA; Department of Horticulture, Washington State University, 303c Plant Sciences Building, Pullman, WA 99164-6414, USA; Department of Horticulture, Washington State University, 303c Plant Sciences Building, Pullman, WA 99164-6414, USA

## Abstract

The unprecedented volume of big data being routinely generated for nonmodel crop species, coupled with advanced technology enabling the use of big data in breeding, gives further impetus for the need to have access to crop community databases, where all relevant data are curated and integrated. Funding for such databases is, however, insufficient and intermittent, resulting in the data being underutilized. While increased awareness of the importance of funding databases is important, it is practically necessary to find a more efficient way to build a community database. To meet the need for integrated database resources for various crop genomics, genetics, and breeding research communities, we have built five crop databases over the last decade using an open-source database platform and software. We describe the system and methods used for database construction, curation, and analysis protocols, and the data and tools that are available in these five crop databases.

**Database URL**: The Genome Database for Rosaceae (GDR, www.rosaceae.org), the Genome Database for Vaccinium (GDV, www.vaccinium.org), the Citrus Genome Database (CGD, www.citrusgenomedb.org), the Pulse Crop Database (PCD, www.pulsedb.org), and CottonGen (www.cottongen.org)

## Introduction

Technological innovation has propelled crop science into an era of discovery and application driven by “big data,” where scientists routinely generate and analyze larger, ever more complex, and new types of genomic, genetic, and breeding datasets for both model and nonmodel crop species. The size and diversity of this big data are unprecedented, from numerous whole genome datasets within a species, expression data across environments, to phenotypic and genotypic data for tens of thousands of individuals. When the data are organized, annotated, and integrated with other associated data, and made available with supporting tools for browsing, querying, and analysis, significant value is added for researchers. Data become a reusable, enriched critical resource that supports new and accelerated research discovery and application. Community databases managed by experts with crop-specific knowledge and computational skills are the logical medium with which to meet these challenges efficiently. Community databases developed over the last 25 years provide this critical role for many crops (MaizeGDB [1], GrainGenes [2], Solanaceae Genomics Network [3], SoyBase [4], Genome Database for Rosaceae (GDR) [5], and CottonGen [6]).

The scientific and programming expertise required to build biological databases is very costly and is a major hurdle in developing these community databases for other crops with fewer funding opportunities. In addition, the databases need to be able to adapt and accommodate new data and new data types and have appropriate and easy-to-use tools to query and visualize the new data types and associated integrated data. Databases built with customized database code are often hard to modify or extend with incoming new types of data. This makes the databases complex to manage, unable to easily accommodate “big data,” and resource-intensive to leverage for other crops.

Fortunately, the Tripal software system [7, 8] enables the development of biological databases with relatively minimal programming. Available since 2010, Tripal is an ontology-based, open-source toolkit for the construction of online biological databases [9]. Tripal combines the content management system Drupal (https://www.drupal.org) and the controlled vocabulary (CV)-driven biological relational database storage backend Chado [10]. The CV is widely used to describe data types in Chado, making it relatively easy to introduce new types of data. In addition, Tripal V3 has been redesigned to facilitate data sharing through the systematic use of CVs [8]. The use of Tripal, as a result, encourages databases to practice findable, accessible, interoperable, and reusable data principles [11].

Tripal is increasingly being used in building databases with many public genomic and genetic databases using this platform [7, 8]. There are over 30 known public sites built in Tripal and over 130 public instances worldwide tracked by Drupal reports [7]. Since Tripal is an open-source and modular software, site developers can create their own extension modules to share with other Tripal users. Over 40 extension modules are available on the Tripal website (https://tripal.info/) for use by database developers. Some examples include the Tripal BLAST (https://github.com/tripal/tripal_blast/), which provides an intuitive user interface to the National Center for Biotechnology Information (NCBI) Blast+ tool; Tripal MegaSearch module, which allows interactive and customizable query and download [12]; the TripalMap module, which displays genetic map data [13]; the Tripal Analysis Expression module for loading, annotating, and visualizing NCBI Biomaterials and expression data [14]; and the Breeding Information Management System (BIMS) that allows breeders to store, manage, archive, and analyze their public or private breeding program data [15].

Since 2010 we have built six community databases (https://www.nrsp10.org) for specialty crops and cotton using Tripal. Our goal is to provide an integrated web-based community database resource providing access to publicly available genomics, genetics, and breeding data and data-mining tools in the most efficient way. This report describes the system and software that we used and the procedures for data curation, analysis, and data upload as well as the data and functionality of our databases. Our five current databases are the GDR [5], CottonGen [17], the Citrus Genome Database (CGD), the Pulse Crop Database (PCD), and the Genome Database for Vaccinium (GDV). The databases can be accessed at https://www.rosaceae.org/, https://www.cottongen.org/, https://www.citrusgenomedb.org/, https://www.pulsedb.org/, and https://www.vaccinium.org/.

## Hardware

While our databases could be hosted on a single server, they are hosted using a split architecture due to best practices, the scope of crop databases, and the additional websites hosted in our program. The application server, which hosts Tripal and Drupal, has 2 × 8-core Intel Xeon E5-2620v4 Processors, 128GB RAM, and 10TB usable storage. The database server, which hosts PostgreSQL, has 2 × 6-core Intel Xeon Gold 6128 processors, 8TB storage, and 256GB RAM. Ten additional databases and websites are hosted on these servers using the same split configuration.

As a starting point, a single site could be hosted using Intel Xeon E-series processors, 2–3TB storage, and 256GB RAM. Depending on client usage, performance needs, and data stored, it may be necessary to scale up from this minimum recommendation.

## Software used for database construction

### Tripal core module

Our crop databases were built using Tripal version 3.3. The Tripal Web Services module was enabled to allow data exchange with other Tripal sites. For data visualization, Tripal v2 legacy modules were enabled, in favor of the Tripal DS module. The storage backend was Chado version 1.2, installed on a PostgreSQL database (version 12.9). These modules are available at https://tripal.org.

### Tripal extension modules

Thirteen extension modules have been used in building our databases. Table 1 describes the type, name, and brief functionalities of each module. The detailed documentation can be accessed with the source code at https://tripal.readthedocs.io/en/latest/extensions.html.

**Table 1.** Tripal extension modules used in our crop databases

Type	Name	Functionality
Breeding	BIMS—https://gitlab.com/mainlabwsu/bims [15]	A module for an online breeding management system that allows breeders to manage their public or private breeding program data
Data loading/collection	MCL—https://gitlab.com/mainlabwsu/mcl [16] (Jung *et al*)	A module that enables users to upload their biological data in the accompanied templates to the Chado database schema
	Tripal Blast—https://github.com/tripal/tripal_blast	A module that provides a basic interface to allow users to utilize NCBI BLAST+ [19] in the server
	Tripal JBrowse—https://github.com/tripal/tripal_jbrowse [23]	A module that provides integration between Tripal sites and pre-existing GMOD JBrowse [18] instances
	Tripal GenBank Parser—https://gitlab.com/mainlabwsu/tripal_genbank_parser	A module that downloads data from NCBI Entrez Nucleotide database, parses the downloaded flat file, and loads the results into the Tripal/Chado database
Searching	Mainlab Chado Search—https://gitlab.com/mainlabwsu/chado_search (Jung *et al*. 2017)	A module that enables advanced search function for data stored in a Tripal/Chado database. Various search instances are provided for data types such as Gene, Marker, QTL, Map, Trait, Stock, and Organism
	Tripal MegaSearch—https://gitlab.com/mainlabwsu/tripal_megasearch [12]	A module for flexible querying and downloading biological data stored in a Tripal/Chado database
	Tripal Elasticsearch—https://github.com/tripal/tripal_elasticsearch [14]	A module that integrates Elasticsearch to the Tripal site and also allows Cross-Site Querying for searching remote Tripal contents
Visualization/display	Mainlab Tripal Data Display—https://gitlab.com/mainlabwsu/mainlab_tripal (Jung *et al*. 2017)	A module that contains a set of Drupal/PHP templates that organize and extend the default display of the biological data. Supported data types include marker, QTL, germplasm stock, map, project, haplotype block, polymorphism, image, feature, and pub
	TripalMap—https://gitlab.com/mainlabwsu/tripal_map [13]	A module that displays genetic and genomic map data stored in Chado
	Synteny Viewer—https://github.com/tripal/tripal_synview	A module that displays synteny analysis results
Analysis/annotation	Tripal Analysis BLAST—https://github.com/tripal/tripal_analysis_blast	A module that provides a method for loading XML results from the NCBI BLAST program as well as visualization of the results
	Tripal Analysis InterPro—https://github.com/tripal/tripal_analysis_interpro	A module that provides a method for loading XML results from the InterProScan program as well as visualization of the results
	Tripal Analysis Expression—https://github.com/tripal/tripal_analysis_expression [14]	A module for loading, annotating, and visualizing NCBI Biomaterials and expression data

### Non-Tripal software

Non-Tripal software such as JBrowse 1.16.4 [18], BLAST+ 2.12.0 [19], and Pathway Tools 25.0 [20] have been implemented.

### Database schema and data loader

Tripal uses Chado as a backend schema. Genome and transcriptome data are stored in Chado following the documentation provided in Mungall and Emmert [10] and the documentation on the Generic Model Organism Database (GMOD) website. Genome and transcriptome data are loaded using the Open Biological and Biomedical Ontologies loader, Fast Adaptive Shrinkage Threshold Algorithm loader, and Generic Feature Format loader that are provided by the Tripal core module and the loader provided by the Tripal Analysis InterPro module. The synteny data are loaded by the loader provided with the synteny module. The expression data and gene sequence data from NCBI are loaded using the Tripal Analysis Expression module and the Tripal GenBank Parser module.

**Figure 1.**
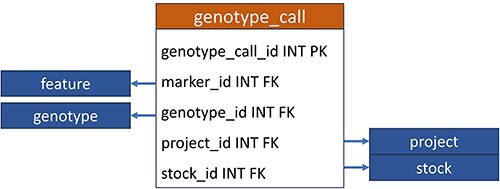
Schematic diagram of how genotype data are stored in Chado. Feature, genotype, project, and stock are existing tables of Chado and genotype_call is a custom table.

The genetic data, as well as genomic and variation data, are stored using various modules in Chado as previously described [21, 22], except the Single Nucleotide Polymorphism (SNP) genotype data, which are stored using the genotype_call table similar to the table described in Sanderson *et al*. [23]. Figure 1 describes how SNP genotype data are currently stored in Chado. This is an update from figure 6 of Jung *et al*. [22]. All the genetic data, as well as phenotype and genotype data, are loaded by the Mainlab Chado Loader (MCL).

## Data curation and analysis

### Whole genome data

We obtain peer-reviewed whole genome data through either publication or direct submission by researchers. The assembly and annotation data are made available to view, download, and search in JBrowse, BLAST, PathwayCyc, Synteny Viewer, Sequence Search, and Gene/Transcript Search pages. Additional analyses provided by our team on the assemblies include computational annotation of predicted genes with the assignment of InterPro protein domains [24] and Gene Ontology (GO) terms [25], as well as homology to known proteins. In detail, the sequences are functionally characterized by pairwise comparison using the BLASTX algorithm against the Swiss-Prot protein database. Information on the top 10 matches with an *E*-value of ≤1E-06 is recorded and stored in our databases together with the sequences. InterPro domains and Gene Ontology assignments are made using InterProScan. The top BLASTP matches for the predicted proteins with the Swiss-Prot and TrEMBL databases are also provided as a downloadable Excel file. Our team also performs synteny analysis to find conserved syntenic regions among the closely related genomes using MCScanX [26] with default settings. PathwayCyc analyses are performed for important genomes using the PathwayTools software ([20]; http://bioinformatics.ai.sri.com/ptools/). The genome assembly and associated annotation file, along with GO terms predicted using InterProScan (https://www.ebi.ac.uk/interpro/download/) and Enzyme Commission terms predicted with DeepEC [27] from predicted proteins, are used to generate the input files for PathwayTools using an in-house Perl script. The PathwayCyc databases for each genome are then built with PathwayTools using the default settings. The built databases are checked for consistency before the final overview is generated and then displayed on our databases.

### Transcriptome data

RNA-seq and dbEST datasets are used to create a reference transcriptome (RefTrans) for each genus or crop and provide putative functions identified by homology to known proteins. The RNA-seq sequences from peer-reviewed publications are downloaded from the NCBI Short Read Archive [28] and subjected to quality control using Trimmomatic (v0.32, default parameters [29]) and custom Perl scripts. The remaining RNA-seq reads are assembled de novo with Trinity (v2.6.6 [30]) using default assembly parameters and a minimum coding length of 200 bases. Quality control of the Expressed Sequence Tags (ESTs) includes vector sequence screening (UniVec_Core, ftp://ftp.ncbi.nih.gov/pub/UniVec/) using cross_match [31], removal of tRNA/rRNA/snRNA sequences identified using tblastx [32], and Poly-A tail trimming. The filtered ESTs are assembled using the CAP3 program (P −90 [33]). Bowtie (v2.3.3) [34] is applied to multi-map the RNA-seq reads and ESTs back to the assembled contigs and singlets. The contigs and singlets are clustered into genes using CD-HIT (v4.6.4 [35]) and Corset (v1.0.7; [36]) with default parameters. The longest isoforms greater than 500 nt are selected to represent each Corset cluster and create the RefTrans sequences. The functional characterization of the RefTrans sequences, the homology with other genes, and the assignment of InterPro domains and GO terms are done by the same procedure as described above. Unigene sets are constructed with ESTs as described in Jung *et al*. [37] when RNA-seq sequences are not available. RefTrans are mapped to an appropriate whole genome assembly using the alignment tool “BLAT.” Alignments with an alignment length of 97% and 97% identity are preserved.

### NCBI gene sequences and expression data

Our database team periodically downloads crop sequences from the NCBI nr database [28] using the Tripal GenBank Parser module described in Table 1. All sequences are then parsed for gene, mRNA, coding sequence, 5′ UTR, and 3′UTR features and imported into our databases. Similar to predicted genes from whole genome sequences, genes parsed from NCBI are further annotated by homology to genes in other species, InterPro protein domains, and GO terms. Biomaterial data from NCBI, including expression data available from publications, are imported into our databases using the Tripal Analysis Expression module.

### Genetic map, marker, QTL, phenotype, and genotype data

**Figure 2.**
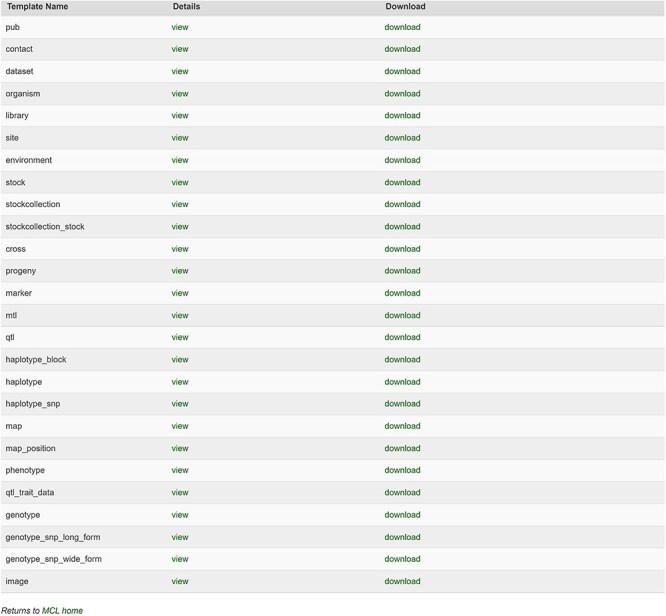
Templates available in the MCL interface. Curators can view and download individual templates or all the templates in one file.

**Figure 3.**
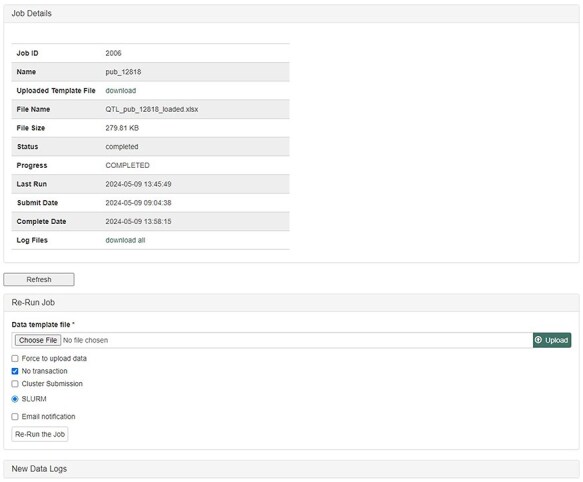
Uploading Job Detail page in the MCL interface. After submitting data in templates, curators can check the progress and any errors.

The database team monitors new peer-reviewed publications on molecular markers, genetic maps, Quantitative Trait Loci (QTLs), phenotype, and genotype data to curate and integrate with other genetic and genomic data. The data in the publication are entered into the templates that accompany the MCL and loaded through the web-based interface of the MCL extension module. Various data templates can be accessed in the curator interface once the MCL module is implemented. In the current version of MCL, templates are available for each of the following data types: db (database), cv (controlled vocabulary), property, pub, library, trait, contact, dataset, image, descriptor, site, stock, cross, progeny, marker, Mendelian Trait Locus (MTL), QTL, map, map position, phenotype, genotype, and haplotype (Fig. 2). Once curators enter the data in the templates, they can upload the data using the web interface. The uploading page shows the status of all the submitted uploading jobs and provides a link to a page, where curators can view the details of each job being uploaded (Fig. 3).

Genetic map data include mapped positions of molecular markers, QTLs, and heritable phenotypic markers, along with information on mapping populations and publications. Marker annotations encompass marker aliases, source germplasm, source descriptions, primer sequences, polymerase chain reaction conditions, literature references, and map positions where available. For SNPs, marker details also include SNP array names, SNP array IDs, dbSNP IDs, alleles, flanking sequences, and probes. QTLs and MTLs data in our databases are curated with aliases, curator-assigned QTL labels, published symbols, trait names, taxa, trait descriptions, screening methods, map positions, associated markers, statistical values, datasets, contact information, and references. Trait names and abbreviations are standardized by curators across all trait loci data entered into the databases to ensure integration with the Trait Ontology (TO)[38], facilitating data integration across databases, organisms, and data types. When terms are not available from TO, we develop new terms for submission.

Our databases also house phenotypic and genotypic evaluation data sourced from publications and/or GRIN [39]. Phenotype data obtained from GRIN or publications are transferred to MCL templates. Details such as associated datasets, phenotypic descriptors, accessions, and phenotypic values are entered into templates and loaded using the MCL loader. Accession names are standardized and compared with those in our databases. Genotype data, such as SNP genotypes, Simple Sequence Repeat genotypes, and haplotype data, are similarly transferred to templates, with marker and accession names standardized before loading into our databases.

## Databases

The GDR, CottonGEN, CGD, GDV, and PCD serve 25 economically, nutritionally, and culturally important crops: fiber (cotton), fruit (apple, apricot, blackberry, cherry, peach, nectarine, pear, plum, raspberry, strawberry, blueberry, cranberry, orange, grapefruit, lime, lemon, tangelo, and tangerine), nuts (almond), pulses (chickpea, fava bean, lentil, pea, and common bean), and ornamentals (apple, cherry, and rose). Grown commercially throughout the USA, the annual US value of production of these crops averaged $26.445 billion between 2016 and 2022 (Table 2). These crops are important for food, feed, and fiber supply and are the economic backbone of many rural communities. These databases are trusted, well established, widely used, community-driven, and highly impactful. They are used in every US state and territory and are the databases of choice of many researchers around the world. Since their establishment (2003 for GDR, 2011 for CGD, GDV, and PCD, and 2012 for CottonGen), the usage of databases has been increasing significantly. Over the past 5 years, they have been collectively cited in 2192 publications, visited by 418 254 users from 185 countries with 7.5 million pages accessed (Table 2).

**Table 2.** The production values of the crops that our databases serve, usage of the databases

Database	Crops served	Average value of production (2016–22) $ billion	2019–23 Usage by community Number of citations
GDR, www.rosaceae.org	Almond, apple, apricot, blackberry, cherry, nectarine, peach, pear, plum, raspberry, rose, and strawberry	14.137	Users = 173 429 Avg. number of countries = 157.4 Visits = 484 134 Pages = 5 730 926 Citations = 1166
CGD, www.citrusgenomedb.org	Grapefruit, lemon, lime, orange, tangelo, and tangerine/mandarin	3.407	Users = 57 386 Avg. number of countries = 143.6 Visits = 96 400 Pages = 678 272 Citations = 132
PCD (formerly Cool Season Food Legume Database), www.pulsedb.org	Beans, chickpea, lentil, and pea	1.454	Users = 40 765 Avg. number of countries = 125 Visits = 55 634 Pages = 296 185 Citations = 54
CottonGen, www.cottongen.org	Cotton	6.345	Users = 129 400 Avg. number of countries = 121.2 Visits = 271 460 Pages = 2 021 507 Citations = 603
GDV, www.vaccinium.org	Blueberry and cranberry	1.112	Users = 27 650 Avg. number of countries = 96.2 Visits = 51 391 Pages = 638 451 Citations = 70
Total	25 crops	26.445	Users = 428 630 Avg. number of countries = 128.7 Visits = 959 019 Pages = 9 365 341 Citations = 2025

### Data types, data integration, and user interface

**Figure 4.**
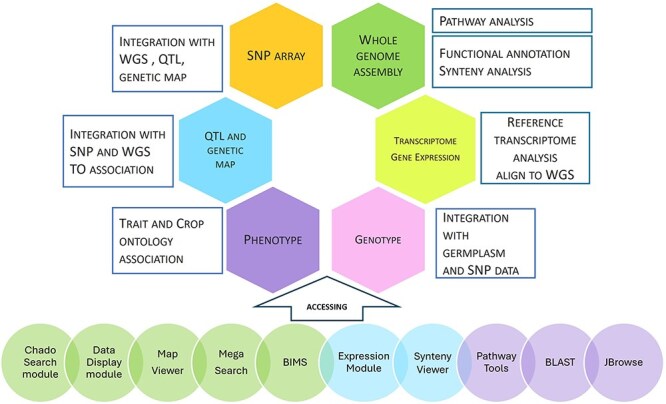
Data types, analysis, integration, and user interface. The first five circles: Tripal modules developed by our group; the next two circles: Tripal modules developed by other groups; the last three circles: non-Tripal software.

Our databases contain whole genome assembly, genes/mRNAs, genetic markers, genetic maps, QTLs, MTLs, phenotype, genotype, haplotype, and publication data. Figure 4 summarizes the data types, data analysis, data integration, and user interface in our databases. Data integration is achieved through various analyses and manual curation. Data with sequences, such as transcripts, markers, and individually cloned genes, are integrated with whole genome data by sequence alignment. Genetic map and whole genome data are integrated when SNPs in whole genome data are used in genetic maps. QTLs are also integrated with whole genome data when the colocalizing markers are aligned to the whole genome. Phenotypic descriptors and trait names of QTLs and haplotype blocks are integrated by associated Trait Ontology. Genotype data are integrated with whole genome data by the markers. Genotype data and phenotype data are also integrated by germplasm. Additionally, genomic regions from related species are integrated by synteny analysis. All the data that are associated with the genomes of one species can be transferred to another species through synteny data. Orthologs identified by the synteny analysis between multiple genome assemblies of the same species represent the same genes, serving as important data integration for users.

Over the years, new types of data and metadata have been added to our databases without changing the underlying database schema due to the ontology-driven and modular nature of Chado. For example, haplotype data and SNP array data, as well as the association of Trait Ontology terms with various data types, such as markers, germplasm, and QTLs, were added without drastic schema changes.

**Figure 5.**
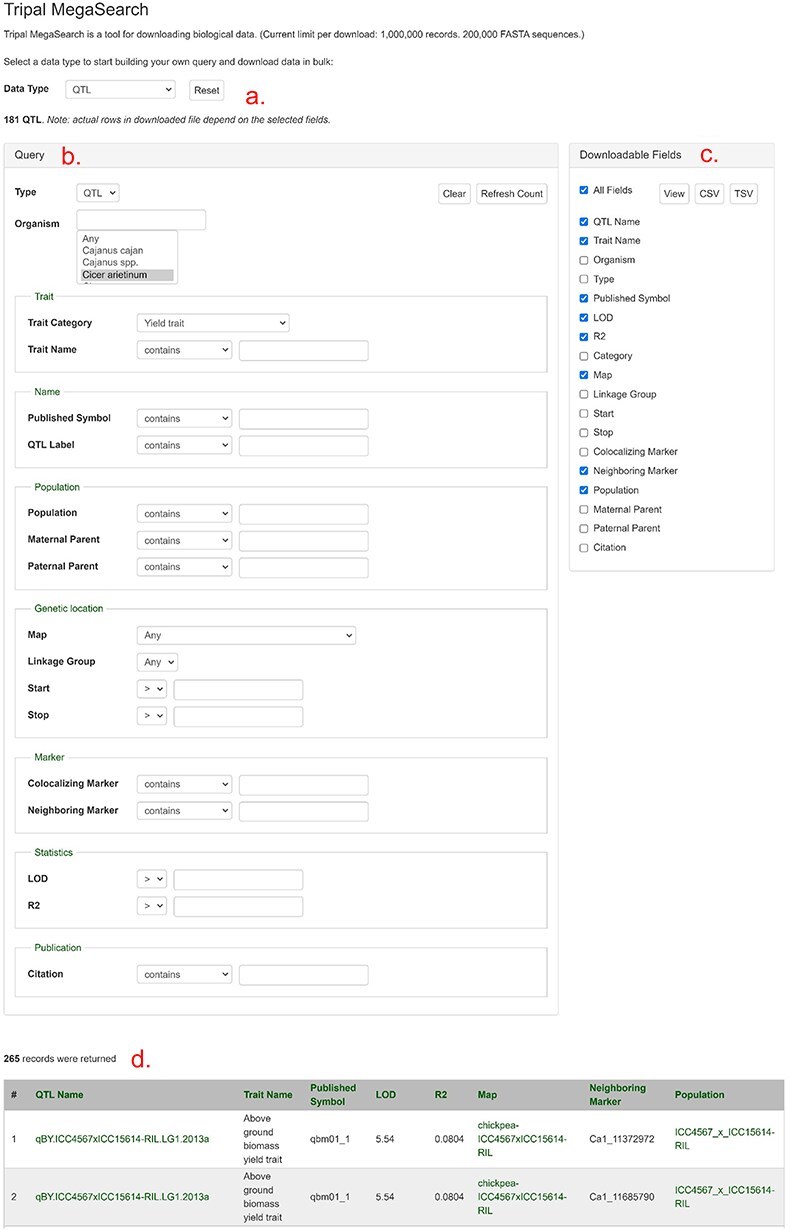
An example MegaSearch page. (a) Data type section where users can choose data type. (b) Query section that provides a query form that allows users to perform complex queries using various metadata as filters. (c) Downloadable Fields section where users can choose data fields to view and download. (d) The result table where users can see the data with chosen fields with hyperlinks.

**Figure 6.**
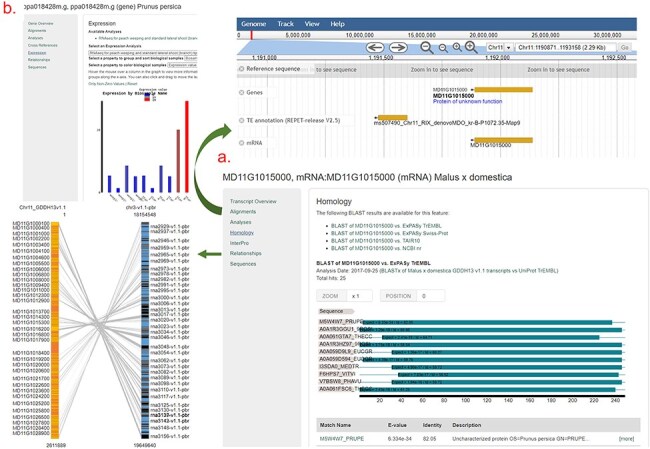
An example gene/mRNA page. (a) Homology section of an mRNA page and JBrowse and Synteny Viewer page that are hyperlinked from Alignments and Relationships sections. (b) Expression section of a gene page that shows a snippet from the Expression Heatmap Viewer.

**Figure 7.**
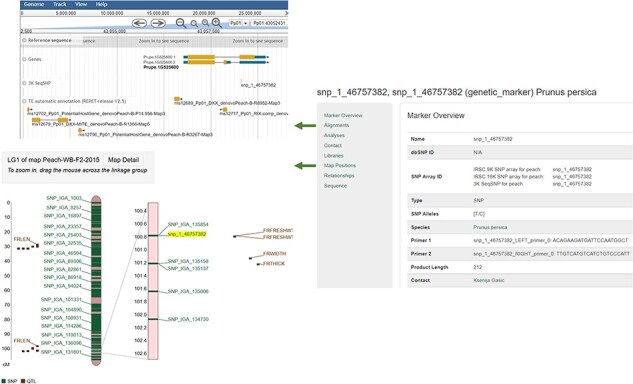
An example Marker page. JBrowse is hyperlinked from the Alignments section and MapViewer page is hyperlinked from the Map Positions section.

As shown in Fig. 4, users can access the data using various search pages and graphic interfaces. MegaSearch (Fig. 5) allows users to search and download various types of data using comprehensive categories and customize the output. Search results for haplotype and genotype data provide a comprehensive view (Fig. 5) and downloading functionality. Individual data pages, such as gene/mRNA page (Fig. 6) and marker page (Fig. 7), allow users to access extensively integrated data. When data are available, graphic interfaces, such as MapViewer, Expression Heatmap, Synteny Viewer, and JBrowse, can be accessed through the link from the gene/mRNA page (Fig. 6) and marker page (Fig. 7). The graphic interfaces can also be accessed directly from the tool menu.

**Figure 8.**
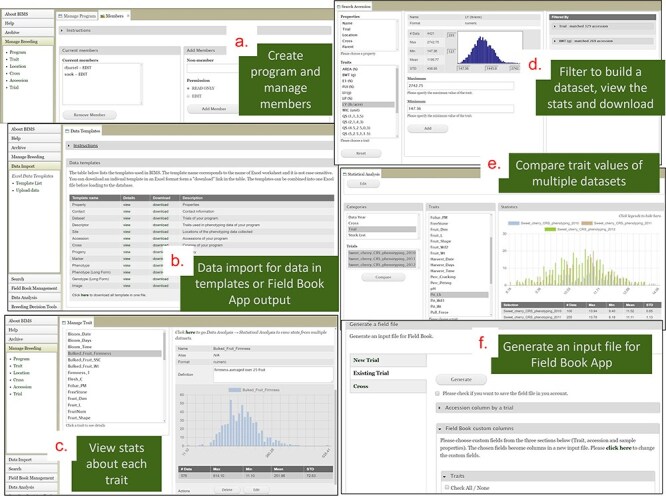
BIMS interface. (a) Manage Program page where users can create a program and add members. (b) Data Import page where users can download data templates and upload their data. (c) Trait section of the Manage Program page where users can view the distribution and the statistical values of the trait values for each trait. (d) Search page where users can filter accessions by various categories including trait cutoff values to build, view, save, and download datasets. (e) Analysis page where users can compare trait values of multiple datasets. (f) Field Book Management page where users can send and receive files to Field Book App using files or Breeding Application Programming Interface.

BIMS is a tool that provides individual breeders with a secure and comprehensive online breeding management system that allows breeders to store, manage, archive, and analyze their private breeding data. Once breeders have their accounts in one of our databases, they can create their own program, add members, upload their data, and edit their data to manage their accession, phenotype, and genotype data (Fig. 8). Breeders can create and maintain multiple programs if they have different crops and/or different projects. For example, breeders can create an additional BIMS program in addition to their own program to manage data from a collaborative project that involves multiple breeders. Since breeders can operate their BIMS program on their own, it does not require any work from the database team except helping them with questions.

**Figure 9.**
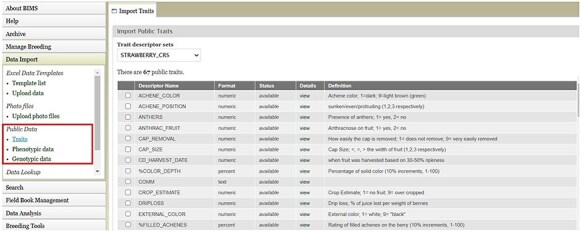
BIMS page in GDR where users can download Strawberry Crop Ontology.

**Figure 10.**
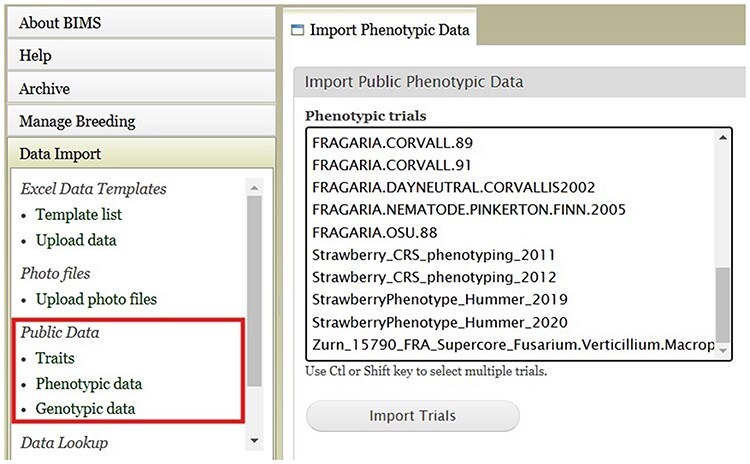
An example BIMS page where users can download public phenotype data.

**Figure 11.**
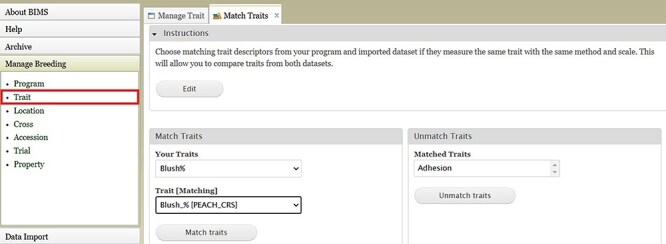
An example BIMS page where users can match trait descriptors from their datasets and imported datasets.

BIMS, when implemented in community databases, can facilitate data standardization and reuse. BIMS users can download community-standard crop ontologies to use as trait descriptors for their private programs In GDR-BIMS, the Strawberry Crop Ontology—developed by the GDR team in collaboration with wider communities—is available (Fig. 9). Similarly, the Blueberry Crop Ontology, developed by community [40], is also available in GDV-BIMS In addition, when public genotype and phenotype data are available in the database, breeders can import them into their private BIMS program. Figure 10 illustrates how BIMS users can view and download publicly available phenotype data in GDR-BIMS. Additionally, BIMS provides functionality for merging trait descriptors when identical traits are measured using different trait descriptors across public and private datasets (Fig. 11). This feature enables users to effectively compare trait data across diverse datasets.

## Limitations

There are some limitations to using the Tripal system for building databases. The underlying schema, Chado, is generic and ontology-driven, which can make it nonintuitive for first-time users. However, various data loaders and publications on how to store data are available, as described above. While creating a genome database with Tripal does not require programming, it is advisable for site developers to have strong programming skills in case new tools or customizations are needed for the site.

## Conclusion and future direction

Building and maintaining our crop databases using the open-source Tripal genome database toolkit have saved significant time and effort, allowing more time to be spent on data analysis and curation. Data curation, analysis, and integration that keep up to date with new publications are key to the usefulness of a crop database. This makes using an efficient database system crucial, especially for orphan crops with limited funding. The control vocabulary-driven Chado also enables the database to accommodate new data types, which further reduces the potential cost of restructuring the database schema and interfaces. We plan to add more types of data to our databases, such as gene annotation data, in collaboration with community researchers and other crop databases.

## Data Availability

The five databases we described are publicly available: the GDR (www.rosaceae.org), the GDV (www.vaccinium.org), the CGD (www.citrusgenomedb.org), the PCD (www.pulsedb.org), and CottonGen (www.cottongen.org).
